# Empirical analysis of health-related behaviors among older Hakka adults: a latent class analysis

**DOI:** 10.3389/fpubh.2024.1396684

**Published:** 2024-08-13

**Authors:** Longhua Cai, Lingling Zhang, Xiaojun Liu

**Affiliations:** ^1^Department of Social Medicine and Health Management, School of Health Management, Fujian Medical University, Fuzhou, China; ^2^Department of Epidemiology and Health Statistics, School of Public Health, Fujian Medical University, Fuzhou, China

**Keywords:** older Hakka adults, health-related behavior, latent class analysis, influencing factors, sociodemographic characteristics

## Abstract

**Background:**

Little is known about health-related behaviors of the older Hakka population in China. We aimed to explore the characteristics and correlates of health-related behaviors among older Hakka adults.

**Methods:**

We used data from the China’s Health-Related Quality of Life Survey for Older Adults 2018. Latent class analysis (LCA) defined latent classes of health-related behaviors for 1,262 older Hakka adults aged 60 and above. Generalized linear regression and multinomial logistic regression analysis were used to identify factors influencing the number and the latent classes of health-related behaviors, respectively.

**Results:**

The LCA showed that the latent classes could be stratified as the risk group (14.82%), healthy group (55.71%), and inactive group (29.48%). Sex, age, years of education, current residence, living arrangement, average annual household income, and currently employed were associated with the number of healthy behaviors. Compared with the participants in the healthy group, widowed/others (*OR* = 5.85, 95% *CI* = 3.27, 10.48), had 15,001–30,000 (*OR* = 2.05, 95% *CI* = 1.21, 3.47) and 60,001 or higher (*OR* = 3.78, 95% *CI* = 1.26, 11.36) average annual household income, and currently employed (*OR* = 3.40, 95% *CI* = 1.99, 5.81) were highly associated with risk group. Additionally, the participants who are widowed/others (*OR* = 4.30, 95% *CI* = 2.70, 6.85) and currently employed (*OR* = 1.95, 95% *CI* = 1.27, 2.98) were highly associated with the inactive group.

**Conclusion:**

This study identified factors specifically associated with older Hakka adults’ health-related behaviors from an LCA perspective. The findings indicate that policymakers should give more attention to older adults living alone and implement practical interventions to promote health-related behaviors among them.

## 1 Introduction

Population aging constitutes one of the most prominent social trends of the 21st century, with health concerns emerging as particularly pressing challenges (1). The older adults are disproportionately prone to health-related problems, such as chronic diseases, disabilities, and cognitive decline (2–4). As is widely recognized, health encompasses not merely the absence of illness but also an optimal state of physical, mental, and societal well-being. The findings of epidemiological studies have demonstrated that adhering to health-promoting behaviors can effectively reduce the incidence of diseases (5). Furthermore, maintaining health-promoting behaviors is conducive to enhancing both physical fitness and overall quality of life among older adults (6). Nevertheless, studies have indicated that Chinese older adults exhibit relatively low levels of health literacy and a subdued adoption rate of health-promoting behaviors (7–10). This observation highlights the need for targeted interventions aimed at fostering health awareness and promoting healthy practices within this demographic group.

Health-related behavior is a primary factor influencing health. Over the past few decades, the impact of health-related behaviors on individuals’ health status has been extensively examined in numerous medical and epidemiological studies (11, 12). Specifically, five behaviors have been identified as major determinants of individuals’ health status: diet, sleeping, physical activity, smoking, and alcohol consumption (13–15). As an example, studies have demonstrated that smoking and sedentary behavior are associated with increased risk of adverse health effects, such as heart disease, hypertension, stroke, cancer, or diabetes (16–18). The importance of health-related behaviors in affecting health suggests that investigating the determinants of such behaviors should be a primary public health goal, since it might help to promote the adoption of healthy behaviors and mitigate the prevalence of risky behaviors. Studies often examine health-related behavior and its influencing factors separately from one another (19, 20), but emerging research confirms that these behaviors co-exist or cluster in most populations (13). Thus, it is necessary to systematically study multiple forms of health-related behavior.

While associations between health-related behaviors have been noted (13), little is known about the clustering of these behaviors nor their relationship with demographic (i.e., sex, age) factors in this population (21–23). Existing studies have often employed regression models to analyze the factors that influence health-related behavior (24, 25). The failure of uni-dimensional measures to capture the clustering of health-related behavior has led to the utilization of more sophisticated methods, such as the latent class analysis (LCA), to identify health-related behavior typologies and influencing factors associated with varying types of health-related behavior (26). LCA is a probabilistic modeling algorithm that allows clustering of data and statistical inference (27). Rather than analyzing each health behavior in isolation, LCA allows for a person-centered approach to understand how a wide range of health behaviors may cluster together to reflect distinct health-related behaviors within a large and heterogeneous population. Xiao et al. used LCA to classify caregivers of adolescents between age from 12 to 18, based on healthy lifestyles and suicidal behaviors (28). Their findings would help to understand the association between health lifestyles and suicidal behaviors. However, no existing studies have utilized LCA to analyze the characteristics of health-related behavior among older Hakka adults.

The Hakka population is a large branch of the Han Chinese, and there are approximately 80 million Hakka people worldwide, of which approximately 50 million live in Guangdong, Jiangxi, and Fujian in China. Hakka is not only a term for a folk family but also defines a set of common cultural beliefs, which have gradually developed over a 1000 years of their existence (29). In the study by Guo et al., older adults in economically disadvantaged areas, especially in rural areas, had a poorer health status (30). Besides economic factors, cultural factors also have a significant impact on health. Tan et al. demonstrated that health varies across ethnic groups (31). Our study site, Ninghua County, Fujian Province, has the dual characteristics of being underdeveloped and having a strong Hakka culture. Therefore, the objective of this study is to explore the latent classes of health-related behaviors among local Hakka older adults and evaluate the influencing factors of health-related behaviors.

## 2 Materials and methods

### 2.1 Study design and participants

This study is a cross-sectional, community-based survey study conducted in Ninghua County, Fujian Province, China, commonly known as the cradle of the Hakka. Data collection for this study was nested in a larger cross-sectional population-based survey named the China’s Health-Related Quality of Life Survey for Older Adults 2018 (CHRQLS-OA 2018) (32). The data source for this study is the same as an article that has previously been published (33). Our potential study subjects were residents aged 60 years or older, had a local household registration, and voluntarily participated in the survey. Those who had a critical illnesses like aphasia, deafness, blindness, paraplegia, etc., had severe mental disorders or had been diagnosed with dementia, or had a history of mental illness were excluded from our target respondents.

### 2.2 Measures

#### 2.2.1 Sociodemographic characteristics

The sociodemographic characteristics examined included: sex, age (60–64, 65–69, 70–74, 75–79, ≥ 80), years of education (0, 1–6, ≥ 7), marital status (cohabitation, widowed/others), current residence (village, town, county), living arrangement (living alone, living with spouse only, living with children, rotation in children’s homes, others), average annual household income (15,000 or lower; 15,001–30,000; 30,001–45,000; 45,001–60,000; 60,001 or higher), currently employed (no, yes). We set the options for the questionnaire of this study with reference to the China Health and Retirement Longitudinal Study (CHARLS)—a nationally representative survey conducted by the National School of Development of Peking University (34).

#### 2.2.2 Assessment of health-related behaviors

Five health-related behaviors were measured in this study. According to the Healthy China Action Plan (2019–2030) and the purposes of this study, health-related behaviors were defined as follows: (1) healthy diet, i.e., participants who self-reported keep balanced diet and have breakfast every day; (2) regular sleep, i.e., participants who self-reported having regular sleep; (3) physical exercise, i.e., individuals who did meet the standard set by the Chinese Center for Disease Control and Prevention (CDC), i.e., doing exercise more than three times per week and at least 30 min per time; (4) smoking, i.e., individuals who were self-reported smoking at least one cigarette per week were defined as smokers, while individuals who have never smoked or have quit smoking were nonsmokers; (5) drinking, i.e., individuals who self-reported drinking more than one time per week were defined as drinkers, and those who never drank in the past or have quit drinking were current nondrinkers.

### 2.3 Analytic strategy

Statistical Package for the Social Sciences (SPSS) version 23.0 (IBM Corporation, Armonk, NY, United States) and Mplus version 8.3 (Los Angeles, CA, United States) for Windows software was used to perform all statistical analysis work. The alpha level was set at 5% to determine statistical significance.

We conducted a four-part analysis. First, we described the sociodemographic characteristics and the distribution of health-related behaviors of participants. Second, we not only calculated the total number of these healthy behaviors, but also explored the participants’ latent classes within these health-related behaviors by LCA. The exploratory analysis of latent classes was conducted in strict accordance with the steps of LCA. In the analysis, test indexes for model fit included: Akaike information criterion (AIC), Bayesian information criterion (BIC), and adjusted Bayesian information criterion (aBIC). In general, smaller values of these indexes indicate better model fit. The accuracy of the classification is shown by the Entropy, which takes a value in the range of 0–1, the larger the value, the more accurate the classification. In addition, Lo–Mendell–Rubin (LMR) and bootstrapped likelihood ratio test (BLRT) were used to compare the fitting differences of the latent class models, and if their *p*-values reached the significance level (*p* < 0.01), it indicated that the model with K (the number of freely estimated parameters) classes significantly outperformed the model with K-1 classes models. Third, the generalized linear regression analysis was used to analyze factors influencing the number of healthy behaviors. The coefficients (*β*) with a 95% confidence interval (95% *CI*) obtained from the model were reported. Finally, the multinomial logistic regression models were used to identify factors influencing the latent classes of health-related behaviors. The results were presented as an odds ratio (*OR*) value with a 95% confidence interval (95% *CI*).

## 3 Results

### 3.1 Sociodemographic characteristics and health-related behaviors of the sample

Sociodemographic characteristics of older Hakka adults were shown in Table 1. The age distribution of the participants was relatively even, with the percentage of each age group approximately 20%. Nearly half of the participants had never experienced education, and there were more older Hakka adults with 1–6 years of education (39.70%). More than half of the older adults’ marital status was cohabitation (66.80%). More participants lived in the county (40.41%). Fewer respondents lived alone (8.24%), and 31.70% of the participants lived with their spouses only. Minority of participants (7.92%) have 60,001 or higher Average annual household income. More than half of the respondents (68.54%) were currently unemployed.

**Table 1:** Demographic characteristics of participants (*n* = 1,262).

Variables	Categories	n	%
Sex	Male	613	48.57
	Female	649	51.43
Age (years)	60–64	356	28.21
	65–69	248	19.65
	70–74	227	17.99
	75–79	204	16.16
	≥ 80	227	17.99
Years of education	0	583	46.20
	1–6	501	39.70
	≥ 7	178	14.10
Marital status	Married/ cohabitation	843	66.80
	Widowed/ others	419	33.20
Current residence	Village	478	37.88
	Town	274	21.71
	County	510	40.41
Living arrangement	Living alone	104	8.24
	Living with spouse only	400	31.70
	Living with children	435	34.47
	Rotation in children’s homes	235	18.62
	Others	88	6.97
Average annual household income (CNY)	15,000 or lower	260	20.60
	15,001-30,000	349	27.65
	30,001-45,000	339	26.86
	45,001-60,000	214	16.96
	60,001 or higher	100	7.92
Currently employed	No	865	68.54
	Yes	397	31.46

**Figure 1:**
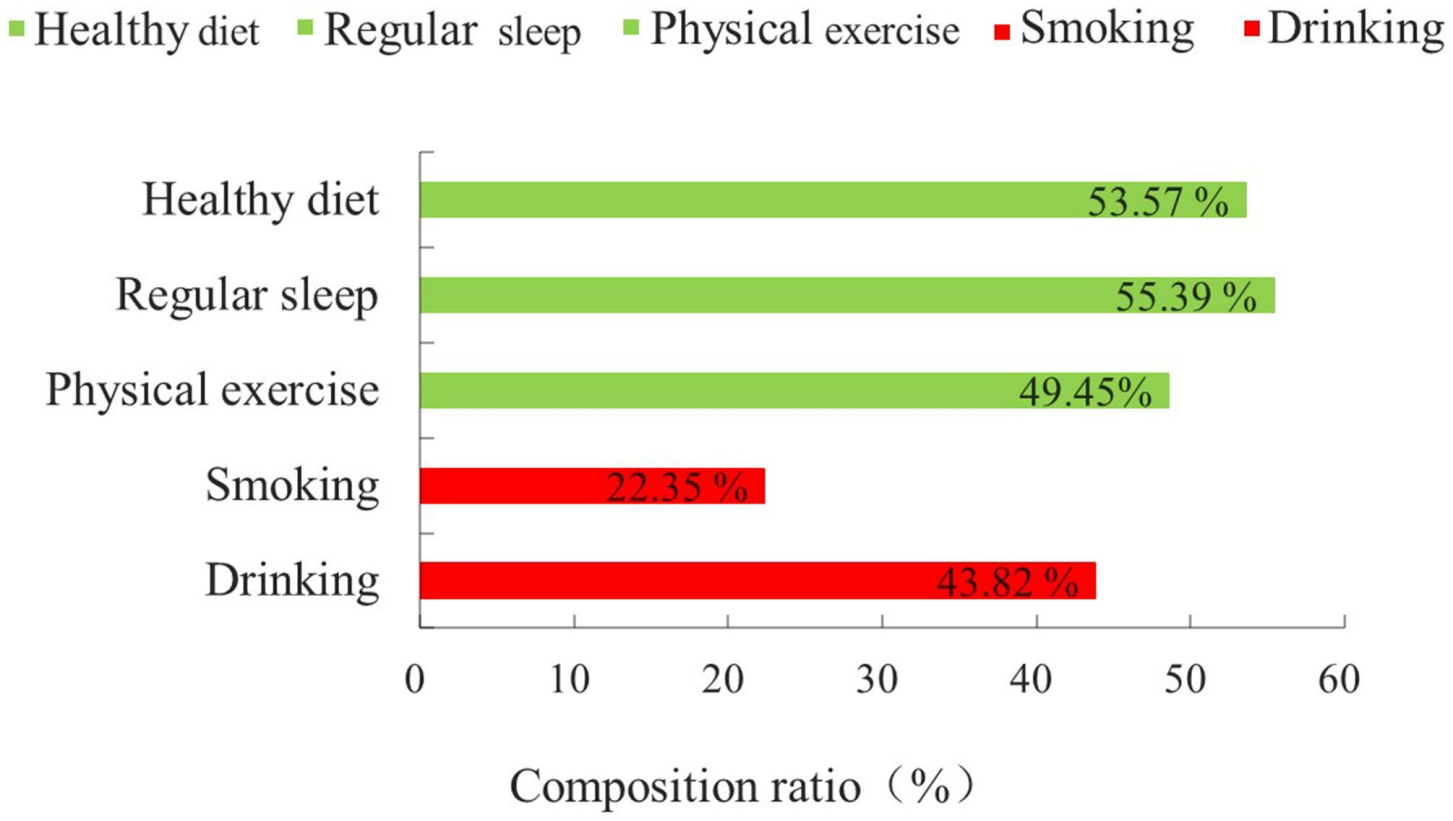
Basic status on health-related behaviors.

Nearly half of older Hakka adults have a health-related behavior of healthy diet, regular sleep, and physical exercise. Among them, regular sleep was the most adopted health-related behavior by participants (55.39%). 282 participants (22.35%) were smoking cigarettes and 553 participants (43.82%) were drinking. The specific results are shown in Figure 1.

**Figure 2:**
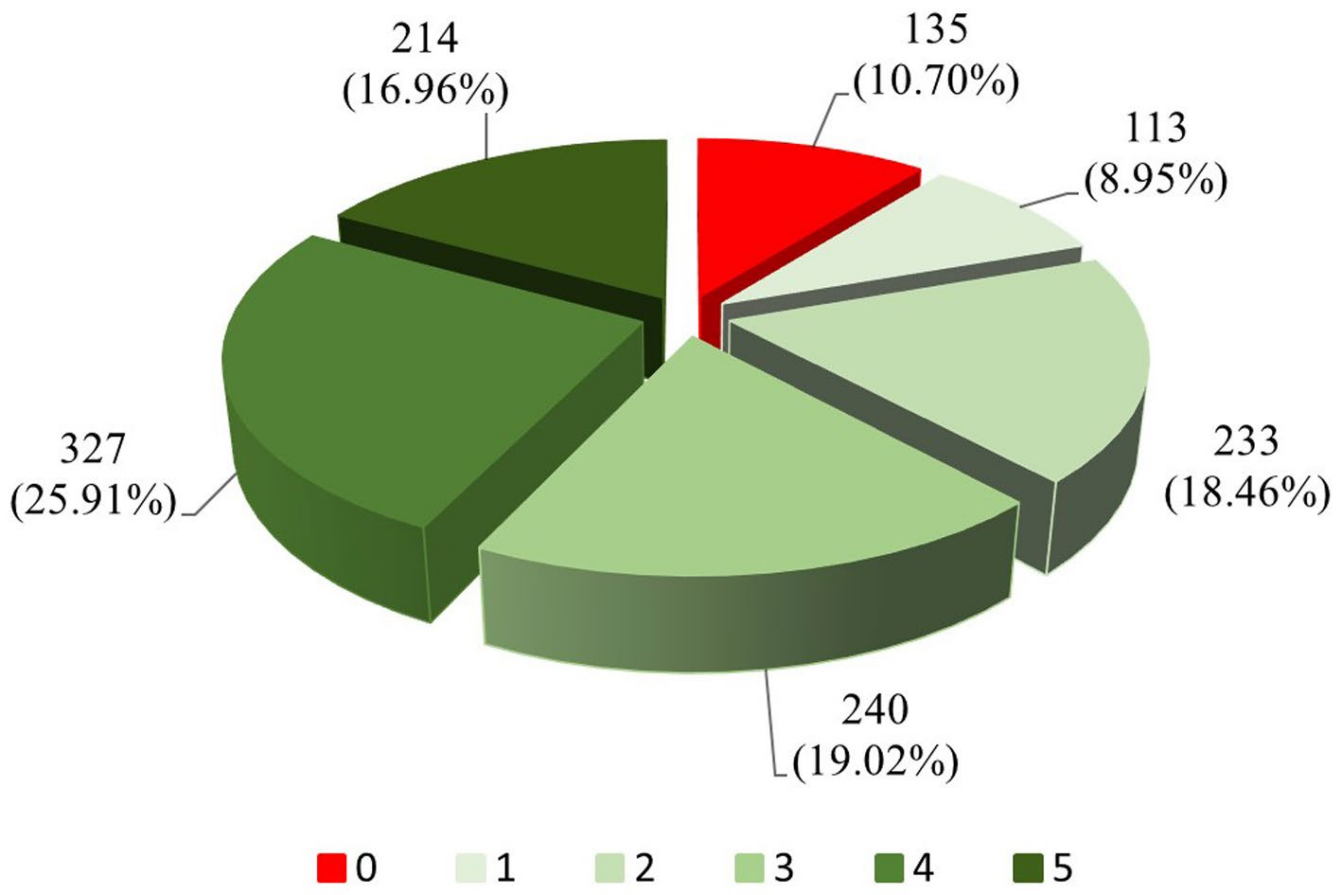
Basic status on the number of healthy behaviors.

The distribution of the numbers of healthy behaviors among older Hakka adults is shown in Figure 2. The largest number of older Hakka adults had four healthy behaviors (25.91%). The least number of participants had only one healthy behavior (8.95%), and 10.70% of the participants had no healthy behavior of concern to this study.

### 3.2 Latent classes of health-related behaviors among older Hakka adults

The model fit for the LCA is shown in Table 2. As the number of model classes increases, the values of AIC, BIC and aBIC decrease until the number of latent classes is 5, the values of BIC and aBIC increase instead of decreasing, and the *p*-value of LMR is no longer significant at this point, suggesting that the model with the number of classes of 4 is more likely to be the best fit. However, in the subsequent analysis, it was found that the probability of one of the classes of the 4-class model was only 0.055. Combined with the actual situation and considering the representativeness and interpretability of each index. Finally, we chose the 3-class model, which was second only to the 4-class model in terms of the degree of fit.

**Table 2:** Comparison of latent class analysis model fit indices.

Number of classes	AIC	BIC	aBIC	Entropy	LMR	BLRT	Class probability
1	8307.410	8333.112	8317.230	–	–	–	1.000
2	7404.433	7460.978	7426.036	0.777	< 0.001	< 0.001	0.597/0.403
3	7200.880	7288.268	7234.268	0.791	< 0.001	< 0.001	0.148/0.557/0.295
4	7168.967	7287.198	7214.139	0.868	< 0.001	< 0.001	0.169/0.055/0.498/0.278
5	7161.341	7310.415	7218.297	0.772	0.214	< 0.001	0.278/0.421/0.020/0.160/0.120

**Figure 3:**
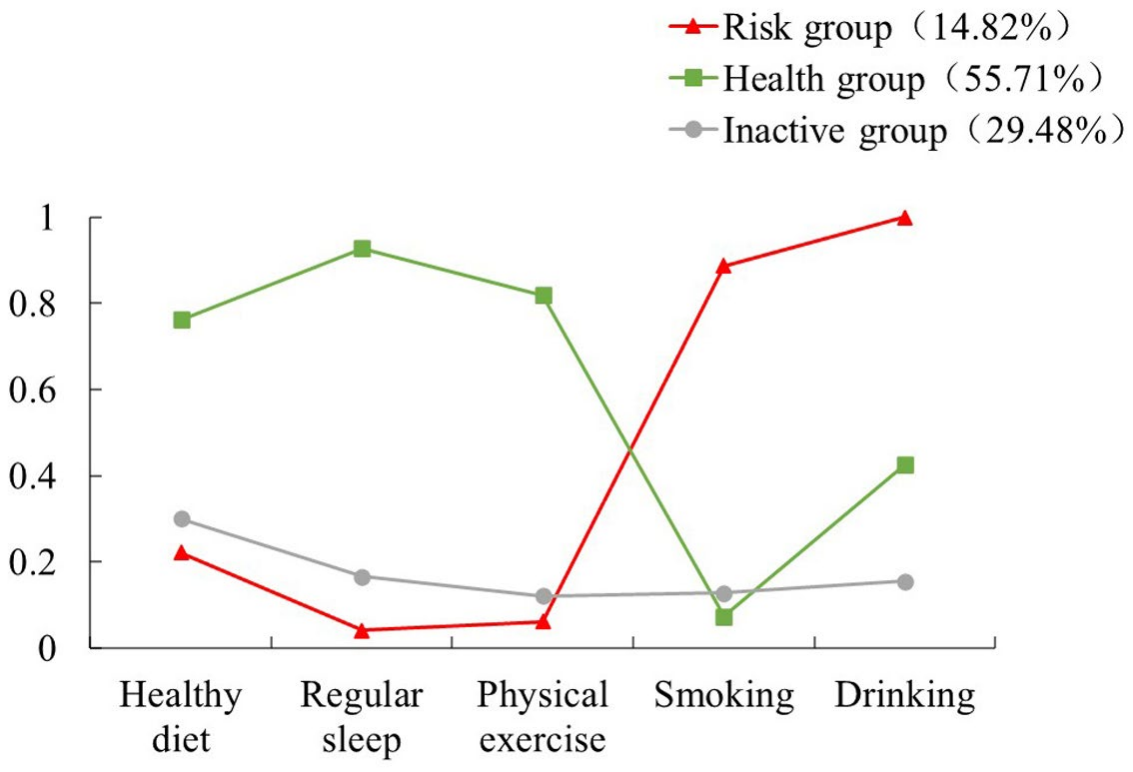
Conditional probability distribution of latent classes.

In order to present the characteristics of each class of the 3-class model more clearly, the latent classes were named according to the distribution characteristics of the conditional probabilities of the latent classes shown in Figure 3. The group in class 1 has a strong tendency toward smoking and drinking, and has an extremely low rate in terms of healthy diet, regular sleep, and physical exercise. Thus, this class is named the risk group (14.82%). Class 2 is distinctly contrary to class 1 in terms of the characteristics of each health-related behavior. The conditional probabilities of each health-related behavior in both classes were nearly symmetrical. Hence, this class was named the health group (55.71%). Meanwhile, the conditional probabilities of each health-related behavior in class 3 were at a low level, so it was named the inactive group (29.48%).

### 3.3 Factors influencing the number of healthy behaviors among older adults

The results of a generalized linear regression analysis of the factors influencing the number of healthy behaviors were shown in Table 3 When compared with the uneducated, those with 1–6 years of education (*β* = 0.27, 95% *CI* = 0.09, 0.45) and those with ≥7 years of education (*β* = 0.31, 95% *CI* = 0.05, 0.58) were more likely to adopt more healthy behaviors. For individuals living with a spouse (*β* = 0.37, 95% *CI* = 0.06, 0.69) or alternating between their children’s homes (*β* = 0.31, 95% *CI* = 0.03, 0.59), a greater likelihood was ascertained in relation to the possession of more healthy behaviors. In terms of age, those aged 70–74 (*β* = −0.24, 95% *CI* = −0.45, −0.02) and those aged 80 and above (*β* = −0.26, 95% *CI* = −0.05, −0.02) were less likely to adopt healthy behaviors. Those whose average annual household incomes were 15,001–30,000 CNY (*β* = −0.21, 95% *CI* = −0.04, −0.02) were also less prone to adopt healthy behaviors as compared to those making less than 15,000 CNY. As for current residence, those who lived in the county (*β* = 0.88, 95% *CI* = 0.66, 1.11) showed greater possibilities in having healthy behaviors.

**Table 3:** Predictors of factors associated with the number of healthy behaviors.

Variables	Categories	*β* (95%*CI*)	*p*-value
Sex (ref = Male)	Female	0.58 (0.44, 0.72)	< 0.001
Age (ref = 60–64)	65–69	0.14 (−0.06, 0.34)	0.184
	70–74	−0.24 (−0.45, −0.02)	0.030
	75–79	0.12 (−0.11, 0.35)	0.302
	≥ 80	−0.26 (−0.50, −0.02)	0.034
Years of education (ref = 0)	1–6	0.27 (0.09, 0.45)	0.004
	≥ 7	0.31 (0.05, 0.58)	0.019
Marital status (ref = Married/ cohabitation)	Widowed/Others	−0.74 (−0.93, −0.55)	< 0.001
Current residence (ref = Village)	Town	0.14 (−0.05, 0.33)	0.152
	County	0.88 (0.66, 1.11)	< 0.001
Living arrangement (ref = Living alone)	Living with spouse only	0.37 (0.06, 0.69)	0.019
	Living with children	0.86 (0.56, 1.15)	< 0.001
	Rotation in children’s homes	0.31 (0.03, 0.59)	0.030
	Others	0.08 (−0.27, 0.43)	0.650
Average annual household income (ref = 15,000 or lower)	15,001–30,000	−0.21 (−0.40, −0.02)	0.031
	30,001–45,000	0.23 (0.00, 0.47)	0.050
	45,001–60,000	0.11 (−0.17, 0.39)	0.427
	60,001 or higher	0.04 (−0.31, 0.39)	0.829
Currently employed (ref = No)	Yes	−0.57 (−0.74, −0.40)	< 0.001

### 3.4 Factors influencing the latent classes of health-related behaviors among older adults

The results of the unordered multinomial logistic regression analysis of the factors influencing the latent classes of health-related behaviors were shown in Table 4. Using the healthy group as a reference, the association between sex, years of education, marital status, current residence, living arrangement, average annual household income and currently employed in the risk group was statistically significant (*p* < 0.05). Those whose average annual household incomes were 15,001–30,000 CNY (*OR* = 2.05, 95% *CI* = 1.21, 3.47) or 6,0001 CNY and above (*OR* = 3.78, 95% *CI* = 1.26, 11.36) were more likely to belong to the risk group than the healthy group. Besides, those whose marital status were widowed/others (*OR* = 4.30, 95% *CI* = 2.70, 6.85) and those currently employed (*OR* = 1.95, 95% *CI* = 1.27, 2.98) were more likely to belong to the group healthy group.

**Table 4:** Predictors of factors associated with the latent classes of health-related behaviors.

Variables	Risk group		Inactive group	
	*OR* (95%*CI*)	*p*-value	*OR* (95%*CI*)	*p*-value
**Sex** (ref = Male)				
Female	0.22 (0.14, 0.36)	< 0.001	1.43 (0.98, 2.07)	0.061
**Age** (ref = 60–64)				
65–69	0.61 (0.31, 1.19)	0.150	0.58 (0.34, 0.98)	0.043
70–74	0.78 (0.38, 1.58)	0.486	0.46 (0.25, 0.84)	0.011
75–79	1.11 (0.52, 2.37)	0.795	0.97 (0.53, 1.76)	0.914
≥ 80	1.84 (0.85, 4.00)	0.124	1.04 (0.57, 1.91)	0.897
**Years of education** (ref = 0)				
1–6	0.77 (0.47, 1.29)	0.327	0.39 (0.25, 0.59)	< 0.001
≥ 7	0.32 (0.13, 0.79)	0.014	0.35 (0.17, 0.73)	0.005
**Marital status** (ref = Married/ cohabitation)				
Widowed/Others	5.85 (3.27, 10.48)	< 0.001	4.30 (2.70, 6.85)	< 0.001
**Current residence** (ref = Village)				
Town	0.96 (0.57, 1.62)	0.891	1.04 (0.67, 1.61)	0.870
County	0.13 (0.06, 0.27)	< 0.001	0.22 (0.13, 0.39)	< 0.001
**Living arrangement** (ref = Living alone)				
Living with spouse only	0.81 (0.31, 2.12)	0.671	1.18 (0.52, 2.68)	0.684
Living with children	0.25 (0.10, 0.61)	0.002	0.44 (0.20, 0.95)	0.036
Rotation in children’s homes	0.57 (0.24, 1.36)	0.206	1.12 (0.53, 2.36)	0.758
Others	1.67 (0.59, 4.71)	0.330	1.52 (0.62, 3.73)	0.356
**Average annual household income** (ref = 15,000 or lower)				
15,001–30,000	2.05 (1.21, 3.47)	0.007	1.38 (0.90, 2.14)	0.143
30,001–45,000	0.95 (0.48, 1.90)	0.886	0.45 (0.26, 0.77)	0.004
45,001–60,000	0.63 (0.22, 1.83)	0.398	0.84 (0.42, 1.67)	0.620
60,001 or higher	3.78 (1.26, 11.36)	0.018	0.53 (0.18, 1.51)	0.234
**Currently employed** (ref = No)				
Yes	3.40 (1.99, 5.81)	< 0.001	1.95 (1.27, 2.98)	0.002

## 4 Discussion

Notably, in our study, we found that the overall health-related behavior status of older Hakka adults is better than that of the average Chinese population. A study reported that 29.40% of older adults in China engaged in mild physical activity in 2018 (35). In contrast, the proportion of older adults participating in physical exercise in the Hakka community is significantly higher, at 49.45%. In addition, the prevalence of smoking among older Hakka adults was 22.35%, which is lower than the national average of 32.10% (32). Bobo et al. found that older individuals residing in areas with low average education levels and limited health-related knowledge were more likely to smoke (36, 37), which contrasts with the findings of this study. This may be attributed to the Hakka people’s higher moral discipline and a deeper understanding of smoking as a detrimental cultural habit, influenced by their philosophy of ‘culture and education’ in family governance. It is noteworthy that the smoking rate among older Hakka adults is not high, approximately 43.82% of them engage in alcohol consumption. This proportion was significantly higher than the national average of 36.30% (38). The results suggest the potential of leveraging cultural influences to control alcohol consumption among older Hakka adults. By focusing on the creative transformation and innovative development of the local drinking culture, we can encourage this population to choose healthier beverages over alcohol.

To the best of our knowledge, this is the first study that used the LCA to identify unobserved subpopulations/classes of health-related behavior of older Hakka adults. This study has confirmed the clustering of health-related behaviors among older Hakka adults. Approximately 80.35% of older Hakka participants reported engaging in two or more healthy behaviors. Significantly, there were 25.91% of older Hakka adults population adopted 4 health-related behaviors. It indicated that promoting the development of additional healthy behaviors among these individuals is likely to yield desirable outcomes. Furthermore, the LCA results demonstrated a clear trend in the classification of health-related behaviors among older Hakka adults, providing additional evidence of its aggregation effect. More than half of the older Hakka adults were classified into the ‘healthy group’, indicating that there is significant potential for further development of health-related behaviors within this population. There were 29.48% of the older Hakka adults were classified as the ‘inactive group’. The individuals in this group did not engage in harmful behaviors such as smoking and drinking, and adopted healthy behaviors like maintaining a balanced diet, having regular sleep patterns, and participating in physical exercise. While a significant proportion of older Hakka adults are committed to sustaining health, they may not be striving for an even higher quality of life.

In terms of sex, older Hakka males were more likely to smoke and drink compared to females, which is consistent with previous findings (39–41). Also, older Hakka females were more likely to participate in physical activity, adopt health-related behaviors, and belong to the ‘healthy group.’ In a study by Hong et al., men were found to have higher health literacy and better health behaviors than women, attributed to the historically strong patriarchal concept in China, where men had more advantages than women in access to economic and educational resources (42). The health-related behavior status of male Hakka older adults is inferior to that of female Hakka older adults, revealing that there is also a degree of “sex-based paradox” in the health-related behaviors of older Hakka adults (43). We found that there were also significant differences in the health-related behaviors of the older Hakka adults in terms of age. The older Hakka adults aged 80 years and above were less likely to have a healthy diet and participate in physical activity than younger Hakka adults aged 60 to 64 years. Our findings are consistent with those of Maynou et al. (44). Our study also found that the older Hakka adults were less likely to practice healthy behaviors, probably because the majority of this population is not well educated and lack of knowledge about health. They are not aware of the role of health-related behaviors in preventing diseases, improving quality of life, and extending life expectancy.

Moreover, our study revealed that marital status and residence patterns were associated with health-related behaviors, which was consistent with prior research (45). Yodmai et al. found that health promotion using family members improved the health-related behaviors of older adults (45). Compared with older adults living with family members, older Hakka adults with marital status of widowed/other were less likely to adopt a healthy behavior, reflecting the importance of family in the development of health-related behaviors for older adults. Indeed, the Hakka adults who did not live alone were more likely to adopt a healthier lifestyle than individuals living alone (46). This may be due to increased loneliness among older adults living alone, which worsens with age and leads to more severe psychological problems. Individuals living with their partners or children tend to have better lifestyle habits as they receive constant care from their partners or children in their daily living environment. Participation and support of family members at home can play a key role in health-related behavior (45). Therefore, it is necessary to provide psychological counseling to older Hakka individuals who live alone or were widowed and to encourage them to adopt healthy behaviors.

In addition, the results of this study showed that economic-related factors such as current place of residence, annual *per capita* household income, and current employment status had significant association with the health-related behaviors. In terms of current place of residence, older Hakka adults living in the county were more likely to adopt healthy behaviors than those living in rural areas. Ohta et al. demonstrated that resources are unevenly distributed between urban and rural areas. Older adults residing in more developed regions have greater access to knowledge about health-related behaviors, facilitated by community organizations (47, 48). The disparity in health literacy between urban and rural areas may have contributed to the poorer health status of older Hakka adults living in rural regions. Regarding current employment status, older Hakka adults who were not employed exhibited a healthier lifestyle compared to those who were still working (49). It has been shown that individuals at lower economic levels are more likely to work longer in their life cycle to earn more income to subsidize their families. The results of this study showed that participants with higher annual household income *per capita* were less likely to adopt health-related behaviors than individuals with low annual household income *per capita*. There are differences in the patterns and roles of the three essential economic factors (socioeconomic, household economic, and personal economic) on the health-related behaviors of the older Hakka population, which deserve further exploration.

## 5 Limitations

This is a cross-sectional study, thus, no causal associations between sociodemographic characteristics and health-related behaviors can be derived from the analysis. Health-related behaviors were self-reported, which may lead to some recall bias and inaccurate results. Besides sociodemographic factors, health-related behaviors are influenced by various profound factors, such as cultural and social factors. Health literacy encompasses individual-level factors and is closely associated with the cultural environment. The Hakka culture is likely to have an impact on the health literacy of older Hakka adults. We overlooked the impact of health literacy and other related variables in this study. Moreover, although we obtained a positive effect of family member companionship on health-related behaviors in older Hakka adults based on the living arrangement, we did not explicitly consider the effect of family clustering on study results. It is necessary to explore the impact of family clustering on health-related behaviors of older Hakka adults from different perspectives in the future.

## 6 Conclusion

In summary, this study provides a comprehensive understanding of the health-related behaviors of older Hakka adults in Fujian, China. The study showed that the health-related behaviors status of the older Hakka adults was generally better than that of the overall Chinese average, but the proportions of excessive alcohol consumers and inactive adults were higher. Females, younger Hakka adults, and those not living alone were more likely to adopt healthier lifestyles. Sex, age, years of education, marital status, current residence, living arrangement, average annual household income, and current work status were the influencing factors for a healthier lifestyle among older Hakka adults. It is recommended that the health education of Hakka older citizens be strengthened, that greater attention should be paid to the adults who are just entering their old age (especially adults between the ages of 60–64), and that the role of family care is fully exploited to promote the development of healthy behaviors among older Hakka adults.

## Data Availability

The raw data supporting the conclusions of this article will be made available by the authors, without undue reservation.
